# Flexible Emotion Regulation: How Situational Demands and Individual Differences Influence the Effectiveness of Regulatory Strategies

**DOI:** 10.3389/fpsyg.2019.00072

**Published:** 2019-02-01

**Authors:** Dorota Kobylińska, Petko Kusev

**Affiliations:** ^1^Faculty of Psychology, University of Warsaw, Warsaw, Poland; ^2^Department of Management, Huddersfield Business School, The University of Huddersfield, Huddersfield, United Kingdom

**Keywords:** emotion regulation, flexibility, context, personality, reappraisal, suppression

## Abstract

The number of studies and theoretical contributions on emotion regulation has grown rapidly. In this article we describe the concept of flexible emotion regulation. We argue that the effectiveness of specific emotion regulation strategies depends on the interaction of the features of a situation and personality characteristics of the individual regulating his/her emotions. We review a few recent theoretical contributions and studies that have attempted to capture some aspects of the flexibility of emotion regulation rather than distinguish between overly adaptive and maladaptive strategies. Moreover, we discuss potential personality determinants of effectiveness of particular regulatory strategies. We claim that further studies should address the interaction of situational and dispositional factors in shaping the effectiveness of particular emotion regulation strategies. So far, situational and personality determinants have been studied rather separately.

## Theories of Emotion Regulation

More and more attention in psychological research is given to the topic of psychological flexibility (Kashdan, 2010). In a fast changing world it seems that to better cope and effectively adapt to changes people need to flexibly choose from a wide range of possible solutions and ways of dealing with difficulties. Our paper taps into the topic of flexibility in the domain of emotion regulation. We propose that flexible emotion regulation is a very effective way of coping and present some preliminary evidence that supports this proposal. Moreover we encourage more research in this area and suggest some possible procedures.

Psychological research shows that emotion, although functional and evolutionary based to increase our chances of survival (Frijda, 1986; Ekman and Davidson, 1994; Oatley and Jenkins, 2003), must be regulated in order to support psychological health and well-being (Jarymowicz, 2008; Aldao et al., 2010) or to help achieve our goals (Aldao et al., 2015). Thus, emotion regulation seems to be a mechanism enabling better coping with environmental demands, so that emotions that are important signals informing about external circumstances or internal states (Jarymowicz, 2002; Jarymowicz and Imbir, 2015) are in fact helpful and advantageous rather than disturbing and disadvantageous.

Emotion regulation is defined in various ways in psychology. Campos et al. (2004) describe a unitary model of emotion and emotional regulation. They argue that emotion manifestation and emotion regulation are indistinguishable interacting processes that do not happen in a sequential manner, but rather appear in parallel and have the same functions. Psychological processes, such as avoiding or seeking situations that are more likely to elicit a particular emotion, can prevent individuals from experiencing the emotion. Accordingly, certain processes, such as appraisal of a situation and self-assessment of one’s regulatory capacity, are responsible for both activating and regulating emotions. However, the majority of researchers claim that emotion generation and emotion regulation are two separate phenomena. Their studies focus on exploring specific regulation strategies. For instance, Garnefski et al. (2001) distinguish nine conceptually different cognitive emotion regulation strategies: self-blame, other-blame, rumination, catastrophizing, putting into perspective, positive refocusing, positive reappraisal, acceptance, and planning. The results of their studies suggest that some strategies are more effective then others.

Other theoretical proposals define emotion regulation as a process aimed at maximization of positive emotions and minimization of negative ones (Larsen and Prizmic, 1999; Wojciszke, 2003), which is called hedonic emotion regulation. Such a definition, however, does not cover the whole spectrum of emotion regulation processes, as sometimes people have instrumental rather than hedonic motives in the regulation of their emotions, for example, to decrease experiencing positive emotions in order to stay focused, or to increase the amount of negative emotions experienced to become more assertive (Gross, 2015a; Tamir, 2016; Ortner et al., 2018). This possibility is taken into account by Gross’s theory of emotion regulation which has recently been highly seminal and dominant.

Gross (2014, 2015a,b) defines emotion regulation as a process by which individuals influence what emotions they have, when they have them, and how they experience and express them. Gross claims that emotion regulation results in changes of the dynamics, duration, and speed of emotion occurrence as well as changes in the consequences of elicited emotion (in behavior, experience and physiology). Emotion regulation can be aimed at reducing, strengthening, or maintaining the experience of either positive or negative emotions depending on the current needs or goals of an individual Gross, 1998, 2002, 2014; Aldao, 2013). In his process model of emotion regulation, Gross describes five families related to the dynamics of the emotional process in which regulation may occur: situation selection, situation modification, attention deployment, cognitive change and response modulation. The first four families of strategies are classified as *antecedent-focused*, because they are employed before the emotional response (Gross, 2002; Ochsner and Gross, 2008, 2014). The fifth family is *response-focused* as it is used after the emotional response has been activated. Moreover, the antecedent-focused strategies are described as more effective (as they change the emotion itself) than the response-focused ones (that change the emotional reaction produced after the emotion has already been experienced).

## Effectiveness of Chosen Strategies of Emotion Regulation

Applying the criteria of frequent use of the strategies in everyday life, a well-explained definition and the possibility of manipulation in the laboratory, Gross and colleagues (e.g., Gross, 1998, 2008, 2014; Gross and John, 2003) focused on two strategies: cognitive reappraisal (antecedent focused strategy) and expressive suppression (response-focused strategy). Most research has compared the effectiveness of these two strategies. Reappraisal is an antecedent-focused strategy that is aimed at modifying the emotional meaning and impact of a situation that elicits emotion (Gross and John, 2003). In contrast, suppression is a form of response modulation and is defined as inhibiting emotional expression (Gross, 1998). As suppression comes later in the emotion-generative process, it does not influence the emotion itself, but rather its outcomes.

Studies have shown that habitual use of reappraisal correlates positively with well-being and negatively with symptoms of psychopathology (Gross and John, 2003; Aldao et al., 2010), while using expressive suppression, is positively correlated with the symptoms of depression and negatively correlated with satisfaction in interpersonal relations (Gross and Levenson, 1993; Srivastava et al., 2009). Moreover, people who habitually use reappraisal experience and express more positive and fewer negative emotions, while those with a tendency to use suppression experience and express less positive and more negative emotions (John and Gross, 2004). What is more, suppression requires self-monitoring and subsequently more cognitive resources (compared to reappraisal), as one has to keep in mind that he or she should suppress as the emotional reaction develops. On the other hand, when reappraisal is done it influences the subsequent emotional process without any further reminders and thus reappraisal consumes fewer resources (Gross, 2002).

According to Gross’s (2014) model, emotions do not need to be regulated or modified all the time but only when they interfere with desired behaviors or goals (Aldao et al., 2015; English et al., 2017). Still, past research on emotion regulation has mostly focused on identifying adaptive or maladaptive strategies in general (Gross and John, 2003; John and Gross, 2004; Gross, 2014, 2015b). Studies on individual differences in tendencies to use reappraisal and suppression, as well as studies on the consequences of the two contrasted strategies activated in experimental research have suggested that reappraisal is more adaptive and “healthier” than suppression (John and Gross, 2004; Mauss and Gross, 2004; Dan-Glauser and Gross, 2015). However, as Troy et al. (2013) argued, this conclusion is incomplete, because no psychological process is always and completely effective and adaptive (Lazarus, 1991, 1993; Grant and Schwartz, 2011). For example, a study by McRae et al. (2012) demonstrated that reappraisal had different effects when individuals were pursuing different goals (either decreasing negative or increasing positive emotions in response to negative stimuli) and using different tactics [ways of achieving the given goals, for example, (a) reality change, (b) distancing, or (c) change of future consequences]. In another study, McRae et al. (2011) described some contextual determinants of frequency of using reappraisal and suppression. Participants in the study conducted at the Burning Man festival reported decreased use of suppression and increased use of reappraisal, compared to typical emotion regulation use at home. This suggests that social context and social situational norms may be important for the choice of different emotion regulation strategies. Although the last study points out the possible situational determinants of the use of specific emotion regulation strategies, it had no experimental design, nor did it give any insight into the topic of strategies’ effectiveness. However, the above mentioned results suggest that the use and adaptability of emotion regulation strategies may depend on the specific context in which it is used (Cheng, 2001; Westphal et al., 2010; McRae et al., 2011, 2012).

## The Importance of Flexibility

Emotion regulation is not aimed at eliminating emotions from our lives, but rather at using them in a flexible manner (Cheng, 2001; Aldao, 2013), using them intelligently (Mayer and Salovey, 1995; Wranik et al., 2007; Matczak and Knopp, 2013; Sìmieja et al., 2014) or understanding them and controlling their influence when this influence is undesired (Kofta, 1979; Kolańczyk and Pawłowska-Fusiara, 2002; Jarymowicz and Kobylińska, 2005; Kobylińska, 2007; Kolańczyk, 2007; Jarymowicz, 2008). The environment we live in is constantly changing. Fixed, inflexible responses, including emotion regulation, are maladaptive in general, and greater flexibility is associated with enhanced adaptation (Aldao et al., 2015) and better coping (Levy-Gigi et al., 2015). As Hollenstein et al. (2013) state, “The success of human evolution has depended on flexible adaptations to shifting environmental demands (...) the development of individuals depends on flexibility as well. The process of development from birth through adulthood can be characterized by a series of challenges that require ever more sophisticated methods of adaptation including learning, self-regulation, and metacognition” (p. 403). Moreover, Kashdan and Rottenberg (2010) examined individual differences in general psychological flexibility looking at the data from experimental research, a diary, a questionnaire and longitudinal studies. The results revealed that general flexibility consistently emerged as a main component of overall health and adjustment.

However, the approach underlying the adaptive functions of flexibility has remained almost entirely theoretical in the emotion regulation domain, with only a few recent examples of studies that tried to capture flexibility of emotion regulation rather than distinguish between overly adaptive and maladaptive strategies. Accordingly, the aim of this article is to explore the research on flexible emotion regulation and to argue that effectiveness of emotion regulation strategies depends on both situational context as well as individual differences in personality like characteristics. We want to show why this topic should be given more attention in psychological studies. First of all, to the best of our knowledge, no studies have addressed the interaction of situational and dispositional determinants of the effectiveness of emotion regulation strategies. Instead, there were two different lines of studies: one focusing on the situational context as a moderator of strategies’ effectiveness and the other one – on personality correlates of emotion regulation (and usually these studies measured either general emotion regulation ability or the tendency to use certain strategies, rather than the effectiveness of different strategies experimentally induced). These need to be joined in order to more fully understand the determinants of emotion regulation effectiveness and the importance of flexible emotion regulation. Secondly, as some researchers have already pointed out (Aldao et al., 2015; Dore et al., 2016), both lines of studies are underrepresented, taking into consideration the huge number of published studies on emotion regulation in general. More insight into the topic of flexibility, coming from research results, is needed. We believe that effective emotion regulation results from certain strategy-situation-personality patterns. Such patterns could be defined on the basis of further studies relying on more complex models of emotion regulation effectiveness. Recognition of such patterns could not only help us better understand the concept of flexible emotion regulation but also serve as a basis for developing psychological interventions aimed at reducing emotion dysregulation and developing adaptable methods of dealing with emotions.

Very recently, Dore et al. (2016) proposed a similar theoretical framework and described emotion regulation as an interaction of person, situation and strategy. They review a number of studies on either situational or individual differences predictors of emotion regulation. However, in the described studies, researchers focused on factors like gender or age as individual differences, rather than on personality characteristics.

## What is Flexible Emotion Regulation?

The flexibility models describe adaptive forms of emotion regulation as involving flexible use of different strategies depending on current situational demands (Cheng, 2001; Bonanno et al., 2004; Kashdan, 2010; Bonanno and Burton, 2014; Koole et al., 2015a). According to some researchers, psychological dysfunction (e.g., affective disorders or borderline personality disorder) may be characterized by deficits in flexibility of used emotion regulation (Rottenberg et al., 2005; Bonanno and Burton, 2014). For example, Bonanno and Burton (2014) claim that emotion regulation strategies are not purely effective and adaptive nor purely ineffective and not adaptive. They report the results of a number of studies that have suggested that a new construct of regulatory flexibility is needed. According to Bonanno and Burton (2014), flexibility is adaptive and lack of flexibility – not adaptive. They point out that future research should focus on finding the best-fit situation-strategy patterns, showing what strategy may be most effective in what situational context. Also, Aldao (2013) and Aldao et al. (2015) encourage research in the important, yet underrepresented domain of emotion regulation flexibility.

We agree that effective regulation in general should be context sensitive and based on a broad repertoire of strategies. Moreover, in line with person-situation models in personality and social psychology (Mischel and Shoda, 1995; Cervone, 2004), we propose that stable individual differences in personality like characteristics may influence the effectiveness of specific emotion regulation strategies applied in different contexts. Thus, we define flexible emotion regulation as the ability to effectively regulate emotions by applying different emotion regulation strategies (chosen from a broad repertoire) in different situations depending on the features of a situation and one’s own personality characteristics.

## Why is Flexible Emotion Regulation Effective?

Taking into account the reviewed theoretical and experimental research, we understand effective emotion regulation as using different emotion regulation strategies flexibly. The flexible use of emotion regulation strategies should be tailored to situational demands and personality characteristics. Such emotion regulation enables meeting the regulatory goals in specific situations (for example, decreasing the strength of negative emotions or staying calm if one has to continue an important conversation with another person) and supports psychological health and long-term well-being. For example, Aldao et al. (2015) propose “…that emotion regulation flexibility is adaptive when it results in an enhanced likelihood of achieving personally meaningful goals (extrinsic, such as losing weight, or intrinsic, such as experiencing calmness”; p. 268). Different situations very often require a completely different approach to deal with emotions effectively and to achieve goals, and there are many different emotion regulation strategies that can be used (for a review, see Gross and Thompson, 2007; Koole, 2009; Webb et al., 2012). At the same time, individuals differ in sensitivity to emotional features of situations and in ease of applying those different approaches skillfully or effectively.

In an extensive review of the emotion regulation literature, Aldao (2013) underlined the importance of many contextual factors that influence the effectiveness of specific regulatory strategies, including personality-like factors, the stimuli used to elicit emotion, the ways emotion regulation strategies are selected and implemented, and the types of outcomes. For example, people with a rich repertoire of emotion regulation strategies might know how to implement adaptive strategies flexibly in response to contextual demands, and thus, might (to a larger extent) benefit from using them (Aldao and Nolen-Hoeksema, 2012a,b; Aldao et al., 2014). Evidence for cultural and social variations in emotion regulation suggests additional personality-like factors that might modulate the consequences of applying specific ways of dealing with emotions (Matsumoto et al., 2008; Mesquita et al., 2014). For example, studies show that in certain collectivist cultures, suppression is not related to poorer psychological functioning and has less negative consequences than usually described in individualist cultures (Butler et al., 2009; Soto et al., 2011).

Several studies have directly addressed the topic of emotion regulation flexibility. However, none of them explicitly tested the interaction of situational and dispositional factors in determining regulation success nor showed direct evidence for the existence of strategy-situation-person patterns influencing effective emotion regulation. They focused on narrower research problems instead, still bringing up some evidence for the effectiveness of flexible emotion regulation.

## Situational Context and Effectiveness of Emotion Regulation Strategies

In the domain of coping (which is very often described as a form of regulating emotions) Cheng (2001) noted that there was little consistency in the use of specific coping strategies across situations, and that a more complete understanding of the coping process should include the examination of the flexible deployment of different strategies in different contexts. Moreover, Bonanno et al. (2004) argued that “successful adaptation depends not so much on any one regulatory process, but on the ability to flexibly enhance or suppress emotional expression in accord with situational demands” (p. 482). In their study, participants’ laboratory task was to enhance emotional expression, suppress emotional expression, and behave normally on different trials. Results supported the flexibility hypothesis and showed that participants who were better at enhancing and suppressing the expression of emotion evidenced less distress in a follow up study. The authors interpret this result as evidence for emotion regulation flexibility.

Troy et al. (2013) described a person-by-situation approach to emotion regulation. In this approach the authors focused on studying reappraisal and argued that as meta-analyses of cognitive reappraisal have shown small to medium effect sizes for predicting outcomes (Aldao et al., 2010; Webb et al., 2012), reappraisal is an adaptive process in many contexts but may not be adaptive in all contexts. According to their proposed person-by-situation approach, the adaptability of different strategies of emotion regulation depends on the situational context in which they are applied. On the basis of research on coping, they assumed that the controllability of a situation might be one of the critical moderators of the adaptability of one’s regulatory efforts. Earlier studies suggested that problem-focused strategies were more adaptive when used in controllable situations while emotion-focused strategies were more adaptive when used in uncontrollable situations (Lazarus, 1993). Troy et al. (2013) hypothesized that reappraisal (which can be considered a type of emotion-focused coping) might be highly adaptive in the context of uncontrollable stress, as in an uncontrollable situation an emotion is the only thing that can be changed. However, when encountering relatively controllable stressors, changing the actual situation (problem-focused coping) might provide more advantages. In this case reappraisal may be less useful or adaptive. In the study, cognitive-reappraisal ability, the severity of recent life stressors, stressor controllability, and level of depression were measured in 170 participants who had recently experienced a stressful life event. Accordingly, the results showed that for participants suffering from uncontrollable stress, cognitive-reappraisal ability was associated with lower levels of depression, whereas for those suffering from controllable stress, higher reappraisal ability was associated with greater levels of depression (Troy et al., 2013). These findings might support the prediction that reappraisal effectiveness may depend on controllability of stressors (although, controllability was not experimentally manipulated) and suggest, in line with our proposal, that “particular emotion-regulation strategies are not adaptive or maladaptive *per se*; rather, their adaptability depends on the context” (p. 2505).

Haines et al. (2016) studied the same strategy-fit-hypothesis in the domain of well-being. Their results showed, supporting the hypothesis, that participants with relatively high well-being used reappraisal more in situations they perceived as less controllable then in situations they perceived as more controllable.

Sheppes et al. (2011) showed some evidence that different strategies may be more or less effective depending on the contexts. In their research the effectiveness of two strategies was examined: distraction (from quite an early stage of emotional process) and reappraisal (from a later stage of emotional process). The results from a series of experiments revealed that in the context of low intensity of emotional situation, people tended to use reappraisal rather than distraction while in the context of high intensity emotional situation, distraction was a preferred strategy. In another set of studies, Sheppes et al. (2014) showed that when stimuli are low in intensity, cognitive demand is low, or when long-term goals are activated, participants had a preference for choosing reappraisal; whereas when stimuli are high in intensity, cognitive demand is high, and when short-term goals are activated, participants had a preference for distraction. These results support the view that strategy choice is context-related. We believe that the effectiveness of the strategies used also depends on the context and we share the opinion presented by English et al. (2017) that “more research is needed to explore the various situational features that may impact emotion regulation strategy use and success” (s. 240).

Bonanno et al. (2004) addressed the topic of flexibility effectiveness. In their study, greater expressive flexibility (reflecting participants’ ability to modify expressions upon command) was associated with better overall mental health and better coping with stressors (Bonanno et al., 2004; Westphal et al., 2010). These results suggest that the ability to switch between different strategies is associated with better regulation effectiveness.

In their review, Bonanno and Burton (2014) focused on coping with stress and emotion regulation, arguing that these are two different domains but are still guided by the same mechanisms of self-regulation. These researchers underline the importance of context in effective emotion regulation and define a concept of context sensitivity as “the ability to perceive impinging demands and opportunities from the situational context as they emerge over and above the normative background of ongoing regulatory concerns and processes and to determine the most appropriate regulatory strategy in response to those demands or opportunities” (p. 594). Sensitivity toward demands and opportunities, as well as threats in the situation serves (according to them) as a crucial component of flexible responding.

## Personality Characteristics and Effectiveness of Emotion Regulation Strategies

Although the studies that addressed the topic of emotion regulation flexibility described above focused on the relation of situational context and emotion regulation effectiveness, we suggest that personality characteristics (relatively stable individual differences) may also be important determinants of the effectiveness of using different strategies.

The existing evidence shows that general emotion regulation ability is related to personality characteristics, for example, to extraversion and neuroticism (Eysenck, 1967; Verduyn and Brans, 2012; Finley et al., 2017), as well as action orientation (Koole and Kuhl, 2007; Koole and Fockenberg, 2015). However these studies do not focus on how personality may shape the ease and effectiveness of using specific emotion regulation strategies.

A few correlational studies show that tendencies to use specific emotion regulation strategies (mostly habitual use of reappraisal and suppression, measured by tests again) correlate with personality characteristics (John and Gross, 2004; Wang et al., 2009; Purnamaningsih, 2017). For example, neuroticism was found to be negatively associated with using reappraisal, extraversion correlated negatively with using suppression (John and Gross, 2004; Purnamaningsih, 2017) and unsafe attachment styles (avoidance and anxiety) were related to frequent suppression use (Gross, 2008). Negative urgency was found to correlate with more using disengagement or reflective emotion regulation strategies (King et al., 2018). In the domain of psychopathology (though it is not the main focus of this paper), Aldao and Nolen-Hoeksema (2012b) showed that certain emotion regulation strategies, such as suppression and rumination, are more strongly associated with psychopathology than other strategies, such as reappraisal and acceptance. In a study by Bloch et al. (2010), clinical populations used suppression more often than non-clinical ones. However, these studies did not address the topic of emotion regulation effectiveness, concentrating instead on subjectively rated tendency to use certain strategies. We believe that there are personality determinants of effectiveness of using different strategies. To test that, we would need studies that test the effectiveness of the strategies’ use, for example long-term effects of applying certain strategies by people with certain personality characteristics shown in longitudinal studies.

Moreover, experimental studies are needed in which different strategies are experimentally induced and personality traits measured. However, in most experimental studies testing the effectiveness of given strategies, personality characteristics are not measured. We believe that this can explain the quite weak effects of theoretically effective strategies obtained in experimental studies (Webb et al., 2012). The effects could be stronger for groups of participants with certain levels of given traits. We think that personality characteristics should be measured as moderators in studies on emotion regulation rather than assuming that random assignment of participants to experimental conditions will control for the effects of personality and other individual differences.

A study by Kobylińska and Marchlewska (2016) can serve as an example of a study that checked how the activated strategy (reappraisal or suppression) interacts with personality in predicting emotion regulation effectiveness. In the study action orientation was measured – one of the trait-like personality characteristics that has been shown to be important for emotion regulation (Kuhl, 1992; Koole and Coenen, 2007; Koole et al., 2015b). From an action control perspective, people with a high level of action orientation have better implicit emotion regulation, which may result in a better fit of their employed strategies to the situation at hand. Action orientation interacted with an activated emotion regulation strategy in predicting regulation outcomes (Kobylińska and Marchlewska, 2016): in a situation requiring emotion regulation (eliciting negative state) suppression was more effective than reappraisal in participants with a low level of action orientation. Thus the strategy, described as rather maladaptive and non-effective, may in fact have better consequences when used by people with certain personality characteristics – here participants low on action orientation. This supports our way of thinking showing that a specific strategy may be more effective for people with a certain level of a trait then for those with a different level of the same trait.

Some indirect evidence of how individual differences may affect the effectiveness of different emotion regulation strategies comes from research on personality determinants of well-being described in the positive psychology literature. Experiencing positive emotions is, for example, a good way to enhance long-term happiness for people with high levels of extraversion and low levels of neuroticism but less effective for those with low levels of extraversion and/or high levels of neuroticism (Pavot et al., 1990; Tamir, 2009). It shows that certain strategies for strengthening well-being (for example, experiencing more positive emotions) are not equally effective for people with different personality traits.

There are also personality characteristics reflecting the ability to implement the proper strategy depending on appraisal of features of a given situation (e.g., Cervone et al., 2008). One such characteristic is emotional intelligence (Pena-Sarrionandia et al., 2015). In general, people with a high level of emotional intelligence should employ strategies that are most effective in given situations. Research shows that working memory capacity may interact with emotional intelligence predicting emotion regulation outcomes (Salovey et al., 2010). Thus, hypothetically, strategies requiring more cognitive resources may be less effective in people with low working memory capacity, even if their level of emotional intelligence is high. To test that we would need an experiment testing the effects of instructing a given strategy either under working memory load or without it and measuring emotional intelligence to see if it moderates the effectiveness of the strategy in those different conditions.

Effectiveness of using different strategies may also depend on dispositional sensitivity to emotional cues (Bonanno and Burton, 2014). For example, according to some studies, depressed individuals are more reactive to sad material (Rottenberg et al., 2002, 2005) and less reactive to positive cues (Treadway and Zald, 2011; Romer Thomsen et al., 2015). At the same time, studies show that emotions evoked by stronger negative stimuli are better regulated by distraction, while those evoked by mild negative stimuli – by reappraisal (Sheppes et al., 2011). Considering these two things together, we may predict that a depressed person would more effectively regulate sadness induced by a movie by using distraction, while for a non-depressed person, the same film may induce sadness that would more effectively be regulated by reappraisal. To test that we should experimentally check whether the level of depression (measures by a questionnaire) moderates the effectiveness of reappraisal and distraction induced experimentally. Thus, measuring a particular individual difference and putting it in the model would give us more detailed knowledge about effective emotion regulation and more understanding about what method works for whom. We could observe stronger effects of reappraisal in participants with low level of depression and stronger effects of depression for those with higher depression level. When we analyze data without the level of depression as a moderator, we may observe weak effects of both strategies for the whole group of participants but this will not reflect the truly effective methods for the mentioned subgroups.

Flexible emotion regulation may also be related to trait-like cognitive flexibility (Stetzel et al., 2013; Goschke and Bolte, 2014). There are studies showing that executive functions, such as emotional updating ability, influence emotion regulation strategies’ effectiveness (Pe et al., 2013, 2015).

Summing up, there are only a few empirical examples of how personality characteristics may shape the effectiveness of different emotion regulation strategies. However, these studies did not test the emotion regulation flexibility hypothesis directly. We believe that more studies are needed to show that personality characteristics predict the effectiveness of different strategies in a different way. Strategy 1 may be more effective when used by Person with personality characteristic A than when used by Person with personality characteristic B, and the opposite with Strategy 2. For example, depending on the level of neuroticism, two different people may experience a different level of stress or negative emotions in the same situation and thus different strategies may be useful for them. As Sheppes and colleagues suggest (Sheppes et al., 2011), distraction is a better strategy when used in highly negative situations, while reappraisal is better in mildly negative ones. Thus, for a highly neurotic person, who experiences more negative emotions even in a mildly negative situation distraction may be an easier or better strategy then reappraisal.

Moreover, as we argue, personality may interact with situational contexts in predicting the effects of emotion regulation. A person needs to be flexible in his/her use of emotion regulation strategies across situations and knowing his/her personality characteristics, he/she may be more amenable to some strategies than others. Thus, strategy 1 may be effective when used in situation 1 by Person A, but not in situation 2 or not when used by Person B. For example, distraction may be an effective strategy when used by a highly neurotic person in a mildly negative situation, but not in a strongly negative one nor used by a person who is low on neuroticism. These, of course, are still hypotheses that need to be further tested.

## Conclusion

Emotion regulation supports psychological health and well-being, as well as helps to deal with negative life events and stress (Gross and John, 2003; Aldao et al., 2010; Troy et al., 2010, 2013; Luhmann et al., 2012; DeSteno et al., 2013; Schwager and Rothermund, 2014; Koole et al., 2015a). Emotion regulation deficits are present in many psychological disorders (see, for example, Werner and Gross, 2010; DeSteno et al., 2013) and, therefore, effective psychotherapy, as well as prevention methods should include strengthening emotion regulation abilities. Better knowledge about which emotion regulation strategies are adaptive in which situational contexts and what strategies can successfully be used by people with different personality characteristics would help to find the best ways to regulate human emotions. Success in emotion regulation has many adaptive outcomes and correlates, such as: better psychological health, increased well-being, better social functioning, better coping with stressful life events, and even school or job success (Salovey et al., 2010). There is a growing consensus that flexible emotion regulation is crucial for prevention and treatment of different affective disturbances that are present in many affective disorders (e.g., Kashdan, 2010; Bonanno and Burton, 2014; Aldao et al., 2015; Koole et al., 2015a). Knowing more about situational and personality determinants of the effectiveness of specific emotion regulation strategies, we could plan interventions that may be better suited for helping specific individuals dealing with emotions in specific contexts. Such interventions, should concentrate on training the broad repertoire of strategies and showing the conditions under which they are effective or not. Additionally, training should involve becoming more aware of ones own traits and preferences that may influence the use and effectiveness of the strategies. It seems that this type of interventions would be more effective in strengthening well-being and reducing the negative effects of stress. Results obtained by Troy et al. (2013) suggest that interventions should focus on both strengthening regulatory ability and learning to use specific emotion regulation strategies in context-appropriate ways. We believe that such interventions should be aimed at teaching flexibility and developing knowledge about specific situations and their demands, as well as developing broad repertoires of strategies (training many different emotion regulation strategies), rather than training one specific strategy.

## Author Contributions

DK (75%) was responsible for the literature search, drafting the paper, working on the final version, and the final corrections. PK (25%) was responsible for consulting the first draft and working on the final version together with DK.

## Conflict of Interest Statement

The authors declare that the research was conducted in the absence of any commercial or financial relationships that could be construed as a potential conflict of interest.
